# Somnoform, an Anæsthetic

**Published:** 1903-04

**Authors:** 


### Reviews of Dental Literature.

Somnoform, an Anæsthetic.—“ Somnoform,” an anæsthetic introduced by Dr. G. Rolland, of Bordeaux, at a meeting of the French Association for the Advancement of Science, held at Ajaccio, in 1901, seems to meet, to a notable degree, the requirements of dentistry. A paper dealing further with somnoform, read before the same society at Toulouse, in August last, published in *L'Odontologie,* October 30, 1902, is summarized as follows:

Up to the time of the meeting, he had administered somnoform “ seven or eight hundred” times in his own practice, for short operations.

To the question, put at the meeting of the year before, as to the safe duration of the anæsthesia, he replies with his experiments.

*On Animals.*—He has *somnoformed* a dozen guinea-pigs for an hour, without ill effect.

"Every one knows the vulnerability of these little animals to an anæsthetic. I have wished to see also what would be the extreme effect of an anæsthesia of an hour, repeated day after day, and of my guinea-pigs some die at the end of four, five, six, eight days."

He had anæsthetized rabbits, dogs, and cats for one, two, three, and four hours without any fatality.

Finding a cat in the streets mortally injured, he decided to kill it with somnoform, after experimenting with it. He had kept the cat under the somnoform eight hours, when he was called away. It took, at the end of that time, four minutes and twenty-seven seconds, twelve cubic centimetres of somnoform, and "an absolute blockage of the respiration." Just what is meant by this last phrase seems doubtful.

*On Man.*—Its administration is followed by a number of manifestations, usually easily discernible, but sometimes following each other so rapidly that one must be accustomed to the use of the drug to recognize them. They are, first, a fixed gaze, or loss of expression of the eye. Sometimes, though rarely, "contrary to that which happens with ethyl chloride, the pupil is dilated." Generally, at this moment, the patient is in a state of semi-consciousness, but is oblivious of pain.

Second, stage of contractions. The behavior of the patient in this stage varies. There may be muscular contractions or clonic spasms. The arm, when raised, remains raised and contracted. The degree of consciousness is much diminished, but the reflex or medullary actions are aggravated, though the sensorium registers nothing. The patient may make an outcry, perhaps, but the anæsthesia is complete.

Third, stage of relaxation. The eyelids close, the limbs are motionless, the raised hand falls. Anæsthesia is complete. This is the moment to operate. Consciousness and all the reflexes, except the conjunctival, are lost.

Fourth, loss of conjunctival reflex. It is necessary to push the anæsthesia a good deal further to get this effect. There may be, at this time, an external or an internal strabismus; the eye may roll up or down. The pupil remains contracted or normal.

Fifth, dilatation of the pupil. With a very large dose of somnoform Dr. Rolland has seen this symptom in animals, and occasionally in patients undergoing surgical operations. In such cases it is only necessary to let the patient take one or two breaths of air for the pupil to regain its normal size.

Sixth, arrest of respiration. Though he has never seen this in the human, he has produced it in the lower animals. Respiration stops in inspiration. In such cases it has only been necessary to give the animal pure air to see respiration start again; in others, tickling of the nose, or the throwing of a few drops of water on the body, or, finally, artificial respiration, has been necessary—this last sometimes after two minutes of cessation.

"Dostre and Morat pretend that the suspension of the respiration determines the fatal arrest of the heart. This opinion, it seems to me, goes beyond the limits of truth. The arrest of respiration is not a cause, but a stage of a process the termination of which is arrest of the heart and death.

"One does not cause the other, and it is quite rare, if one is watchful, that the arrest of respiration is followed by the arrest of the heart.

"In this I am in accord with all the physiologists and with Lauder Brunton, president of the Hyderabad Commission. When there is a failure of respiration, the centre that controls respiration is paralyzed before that which controls the heart, oftentimes long enough before for one to avert the danger.

"The commission experimented upon one hundred and seventy-one dogs and proved that the arrest of respiration precedes the stoppage of the heart by one to eleven minutes.

"In the case of the cat I killed with somnoform, my only experience, the heart beat for six minutes after the respiration ceased."

Seventh, arrest of the heart. "This last stage need not be reached unless the centres are overcome by a tremendous intoxication with the drug.

"Naturally, all the shades of difference in these seven stages are not seen in all cases.

"The statement that I wish to make is that in more than ten thousand cases I have not seen the least unfavorable symptom in the initial stage, and that I have seen but one case of nausea, with a tendency to fainting, in the case of a person predisposed, after an operation of twenty minutes where I had administered a large dose of somnoform. These symptoms occurred two hours afterwards, during the period of elimination. The majority of patients arise immediately after the operation, may eat, at once, if need be, or go about their occupations.

" How explain the innocuousness of somnoform in the three periods?

" First. In the initial period: (*a*) because of the absence of constriction; (*b*) to its energetic action upon the arterial tension.

" Second. In the stage of surgical anæsthesia: to the elevated tension maintaining its level.

" Third. In the post-operative period: to the normal condition in which the blood remains."

There follows in the paper sphygmographic tracings, blood-counts, and experiments as to the effect of somnoform when agitated with drops of blood in open and closed tubes.

The patient experimented upon was Dr. Rolland himself, when just recovering from an attack of influenza, and shows that, as stated above, the elevation of blood-pressure at the beginning of anæsthesia is one to two degrees of the sphygmographic scale, is maintained at a level, and at the end of the anæsthesia is followed by a depression about equal to the elevation. The pulse drops from eighty-four to a level of seventy-one, the respiration from twenty-eight to twenty.

*Conclusions as to Blood Changes.*—After an anæsthesia of eighteen minutes there were but slight changes. One notes:

1. A slight increase of polynucleated corpuscles.
2. A slight diminution of leucocytes.
3. A very slight increase of eosinophiles.

He concludes his paper by calling attention to the " dominant fact" that somnoform has been administered so many times without ill effect.

The description of three cases anæsthetized by Dr. Rolland before the society at the same congress may be of interest:

I. M. X., eleven years; inhalations thirty-five seconds; anæsthesia perfect; duration of same, one minute. Difficult extractions of left superior first molar, the crown of which was broken off. The patient felt nothing. Recovery rapid without incident.

II. Mlle. X., twenty years; nervous, very timid, weeping, discussing; did not inhale well, yet, nevertheless, anæsthesia was perfect. Inhalation forty-five seconds, anæsthesia forty. Extraction of left superior central incisor. Patient felt nothing.

III. Mme. X.; nervous, very impressionable; at the beginning of inhalations violent, but short muscular contraction, then vasomotor dilatation of face, finally muscular relaxation. Four difficult extractions. The patient cried out and awoke at the last extraction. She said she had felt nothing, but had heard everything. Inhalations, thirty-five seconds; anæsthesia, fifty-five seconds.

In his comparison of ether and chloroform Dr. Rolland does not mention the danger of cardiac arrest through inhibition through the vagus, at the beginning of anæsthesia with chloroform. It will be noted that this initial stage is affirmed by Dr. Rolland to be wholly devoid of danger. Somnoform seems to be worth watching.—C. P. B.

